# Pan-Cancer Analysis Shows *TP53* Mutations Modulate the Association of NOX4 with Genetic Programs of Cancer Progression and Clinical Outcome

**DOI:** 10.3390/antiox10020235

**Published:** 2021-02-04

**Authors:** Wei Feng Ma, Howard E. Boudreau, Thomas L. Leto

**Affiliations:** Laboratory of Clinical Immunology and Microbiology, Molecular Defenses Section, National Institutes of Allergy and Infectious Disease, National Institutes of Health, Bethesda, MD 20892, USA; howard.boudreau@nih.gov

**Keywords:** NOX4, NADPH oxidase, EMT, p53, TCGA, bioinformatics, Pan-Cancer

## Abstract

Previously, we have shown TGF-β-induced NOX4 expression is involved in the epithelial-to-mesenchymal transition (EMT), a process critical for cancer metastasis, and that wild-type (WT) and mutant (Mut) p53 have divergent effects on TGF-β induction of NOX4: WT-p53 suppresses whereas Mut-p53 augments NOX4 mRNA and protein production in several tumor cell models. We sought to validate and extend our model by analyzing whole-exome data of primary tumor samples in The Cancer Genome Atlas (TCGA). We constructed a Pan-Cancer dataset from 23 tumor types and explored *NOX4* expression patterns in relation to EMT and patient survival. NOX4 mRNA levels increase as a function of cancer progression in several cancers and correlate with Mut-p53 mRNA and genes involved in programs of EMT, cellular adhesion, migration, and angiogenesis. Tumor macrophages appear to be a source of NOX2, whose association with genetic programs of cancer progression emulate that of NOX4. Notably, increased *NOX4* expression is linked to poorer survival in patients with Mut-*TP53*, but better survival in patients with WT-*TP53*. NOX4 is negatively associated with markers of apoptosis and positively with markers of proliferation in patients with Mut-*TP53*, consistent with their poorer survival. These findings suggest that *TP53* mutations could “switch” NOX4 from being protective and an indicator of good prognosis to deleterious by promoting programs favoring cancer progression.

## 1. Introduction

The epithelial-to-mesenchymal transition (EMT) is an important developmental program in which epithelial cells lose their polarity and tight junctions and rearrange their cytoskeleton to promote cell migration [1,2,3]. Although critical in normal development and wound healing, aberrant EMT has been linked to cancer metastasis, and over-expression of EMT drivers, such as SNAI1 and SNAI2, are associated with poor clinical prognoses in cancer [4,5].

The NADPH oxidase (NOX) family of enzymes are generators of reactive oxygen species (ROS) such as the superoxide anion and hydrogen peroxide. The human NOX family includes NOX1-5, DUOX1, and DUOX2 [6,7]. NOX1-4 requires the co-factor subunit p22phox encoded by *CYBA* [8,9]. The widely expressed NOX4 isoform is constitutively active and regulated primarily at the transcriptional level. We and others have demonstrated that transforming growth factor beta (TGF-β)-induced ROS generated by NOX4 promote EMT, cell migration and invasion in different cell culture models [10,11,12,13,14,15]. Our previous work indicated that while wild-type tumor suppressor protein 53 (p53) suppresses TGF-β-induced NOX4-driven cell migration, mutant forms of p53 support NOX4 induction and cell migration in vitro [16,17]. However, the clinical relevance of our findings has not been explored on a broad scale in the context of human primary tumors.

Preliminary immune-histological and gene expression surveys of human primary tumor samples have revealed elevated NOX4 protein or transcript levels relative to adjacent normal healthy tissues in several tumor types [13,18,19]. Animal model studies have suggested roles for NOX4 in tumor metastasis and angiogenesis. For example, tumor metastasis is reduced in a mouse breast cancer xenograft model in which *Nox4* expression was silenced in 4T1 cells [15], and angiogenesis was reduced in carcinogen-induced fibrosarcomas in *Nox4* knockout mice [20]. Other in vitro studies using established tumor lines or primary cell cultures support roles for NOX4 in angiogenesis and tumor cell proliferation or suppression of apoptosis [19,20,21,22,23]. However, there have been no broad-based human primary tumor surveys described to date correlating *NOX4* expression patterns with programs of cancer progression specifically in the context of *TP53* mutation status.

The Cancer Genome Atlas (TCGA) is a repository of molecular genetic data from primary tumor samples across many types of cancer. At the time of writing, TCGA contains 39 cancer projects from 29 primary tumor sites [24]. Recently, an emerging technique was adopted in many studies whereby all cancer types, regardless of tissue origin, were combined into “Pan-Cancer” cohorts as a means to discern common gene expression patterns applicable to all cancers [25,26].

We sought to harvest the statistical power of the Pan-Cancer technique on a large number of clinical samples to explore the relationship between NOX4 and p53 in the context of various genetic programs of cancer progression, i.e., EMT, cell migration, proliferation, angiogenesis, along with clinical outcomes. We found evidence that suggests NOX4 plays a role in several genetic programs of cancer progression including EMT, cell migration and motility, extracellular matrix (ECM) adhesion and angiogenesis, and these relationships are differentially modulated by WT and Mut-p53. The clinical outcome associated with an increase in NOX4 expression is different in patients with WT or Mut-p53, in that increased NOX4 in patients with Mut-*TP53* is deleterious, whereas increased NOX4 is protective in those with WT-*TP53*. Together, we describe gene expression correlates that provide insight into the role of NOX4 in cancer progression, and how *TP53* mutation status could be used to modulate NOX4-dependent cancer-related events.

## 2. Materials and Methods

### 2.1. Data Acquisition and Availability

Normalized RNA-seq gene expression and clinical data from primary tumor samples were retrieved from the Broad Institute TCGA Genomic Data Analysis Center using the R package RTCGAToolbox [27]. These data have been de-identified and are publicly available with no restriction for publication. Data obtained are based upon data generated by the TCGA Research Network (http://cancergenome.nih.gov (accessed on 7 January 2021)) with a run date of “20160128”. Mutation annotation files were downloaded from cBioPortal for Cancer Genomics [28,29]. Mutation data were joined with the gene expression dataset by patient ID and checked for missing data using in-house R codes. Samples with incomplete mRNA profiles or conflicting p53 mutation annotations were excluded from the analysis. The complete analysis, required files, and R scripts can be found at https://github.com/wfma/Ma_2018 (accessed on 7 January 2021), (doi:10.5281/zenodo.1249667).

### 2.2. NOX4 Expression by Cancer Stages

The NOX4 RSEM (RNA-seq by expectation-maximization) values were plotted as a function of the clinical histopathological grade of the samples in each cancer study. “Relative Fold Change” denotes the natural log of RSEM values, which provide better visualization than non-transformed RSEM values. Data sorting and matching were conducted using in-house R scripts. Kruskal–Wallis rank sum tests were performed to calculate the significance of variance and post hoc Dunn tests for multiple comparisons were used to determine the adjusted *p*-values.

### 2.3. Correlation of NOXs with Genetic Programs of Cancer Progression and Macrophage Markers

Markers involved in genetic programs of EMT, migration, adhesion, angiogenesis, adherens junctions, and markers of M1 and M2 macrophages were identified using Gene Set Enrichment Analysis (GSEA) and literature reviews [3,30,31,32,33,34,35,36]. The most common p53 mutations within the DNA-binding domain from residue 101 to 306 were identified using literature reviews and the International Agency for Research on Cancer (IARC) p53 Database R18 [34,35]. Non-parametric Spearman’s rank tests were conducted to calculate the correlation value *ρ*, which indicates the presence of a monotonic relationship in the range of −1 to 1: +1 indicates a perfect positive correlation, −1 for a perfect negative correlation, and 0 for no correlation. Heat maps with unsupervised clustering were generated using the R packages ggplot2 and Superheat. Finally, to compare the *ρ* values, we employed two-tail Fisher r-to-z transformations where appropriate.

### 2.4. Survival Analysis

Patients were first grouped as either having WT-*TP53* or any of the common *TP53* mutants studied. Then, the Kaplan–Meier survival plot function from the survminer R package was used to estimate survival outcome based on NOX4 gene expression [36]. Relative high- and low-expression designations were optimized using the outcome-driven algorithm from the ‘surv_cutpoint’ function in survminer, which provides an RSEM cut-off value that corresponds to the most statistically significant survival difference. Finally, log-rank tests were used to compute the *p*-value.

## 3. Results

### 3.1. Data Retrieval and Dataset Composition

**Figure 1:**
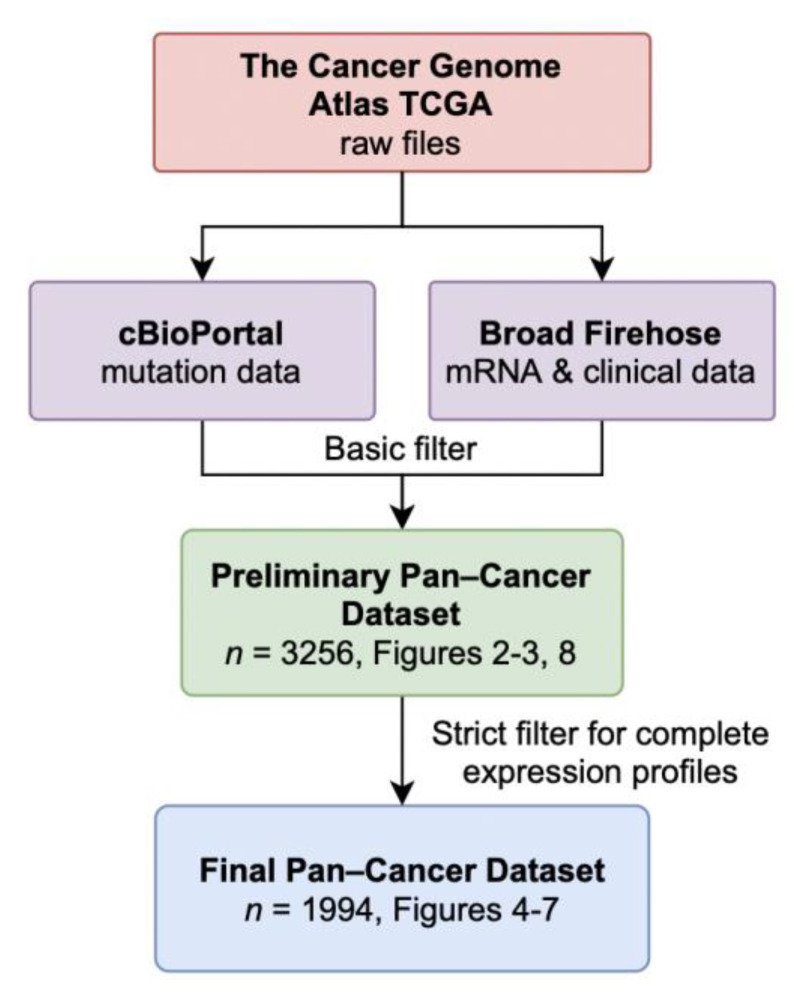
Data retrieval schematic and data composition. De-identified mutation files were downloaded from cBioPortal.org, mRNA and clinical files were retrieved from the Broad Firehose using the R programming language. A basic filtering algorithm checked for conflicts and joined the files into the Preliminary Pan-Cancer Dataset with 3256 samples. A strict filtering algorithm removed samples with incomplete expression files required for the downstream analysis, resulting in the Final Pan-Cancer Dataset with 1994 samples.

From the TCGA database, we downloaded all available gene expression and clinical data, and identified tumor samples with WT-*TP53* and those with common *TP53* mutations within the DNA-binding domain. We excluded patients with *TP53* frameshift mutations, multiple mutations, and patients missing p53 annotation (Figure 1). Samples from all tissue types were pooled together to form a Preliminary Pan-Cancer dataset (Table 1). This dataset includes 2368 samples with WT-*TP53* and 888 samples with Mut-*TP53* for a total of 3256 samples. Although a Pan-Cancer dataset may not reveal tissue-specific gene expression signatures in each tumor, merging sample data from different tissues increases the ability to identify common molecular genetic aberrations that could be generalized to many tumor types [25].

The Preliminary Pan-Cancer Dataset was then selected for samples with complete expression profiles for genetic programs relating to cancer progression, e.g., EMT, resulting in the Final Pan-Cancer Dataset with 1994 samples from 23 different primary tumor sites. Twenty-one studies examined either adenocarcinoma or carcinoma, and two examined sarcomas (Table 1). The most abundant tissue samples were bladder urothelial carcinoma (BLCA, *n* = 294) and brain lower grade glioma, (LGG, *n* = 247). These 1994 samples contain the complete clinical-pathological, gene expression, and *TP53* mutation status data required for the subsequent analyses.

### 3.2. NOX4 Expression Is Increased in the Advanced Stages of Several Cancers

**Figure 2:**
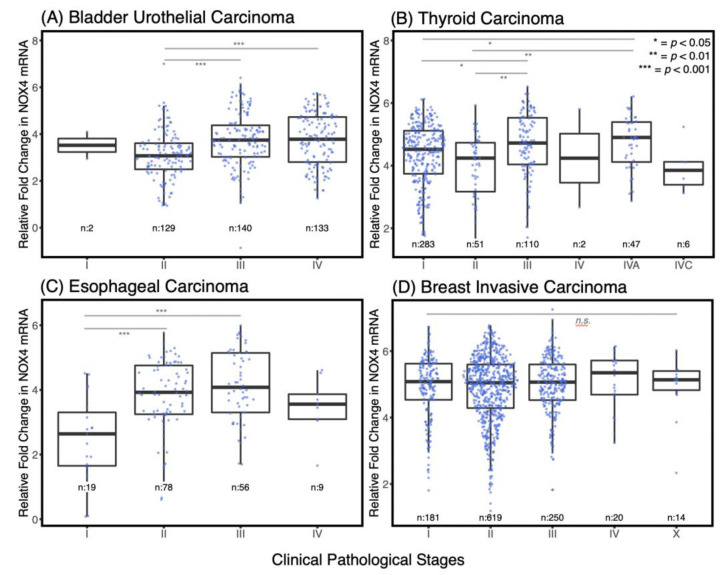
NOX4 mRNA levels are increased in the advanced stages of several cancer types. Samples were grouped by studies and subdivided into clinical stages. Boxplots representing the relative fold change in NOX4 mRNA (natural log of NOX4 RNA-seq by expectation-maximization (RSEM) values) were generated and Kruskal–Wallis rank sum tests along with post hoc Dunn tests for multiple comparisons were used to determine the significance in variance and the adjusted *p*-values. In bladder, thyroid and esophageal carcinoma, NOX4 mRNA levels generally increase as the cancers progress ((**A**–**C**), respectively), although NOX4 remained unchanged in breast carcinoma (**D**). The number of samples is indicated below each bar.

Previously, we have found that overexpression of NOX4 enhances cell migration and wound closure in epithelial tumor cell culture models [11]. Here, we began by exploring whether *NOX4* expression is related to cancer progression by examining NOX4 mRNA levels relative to histopathological stages. Because each cancer has distinct criteria for its clinical grade, we analyzed NOX4 mRNA expression patterns by tumor types and not in a Pan-Cancer manner. We found NOX4 is upregulated as a function of the clinical histopathological grade in bladder urothelial carcinoma, thyroid carcinoma, and esophageal carcinoma, suggesting that NOX4 may be involved in cancer progression (Figure 2). Although NOX4 is not differentially expressed in different stages of breast carcinoma, our previous breast cancer models showed a strong NOX4 influence on cell invasiveness [11,16]. These data highlight the need for a Pan-Cancer cohort in order to discern gene expression patterns that may be applicable to all cancers.

### 3.3. Mut-p53 mRNA Positively Correlates with NOX4 mRNA

**Figure 3:**
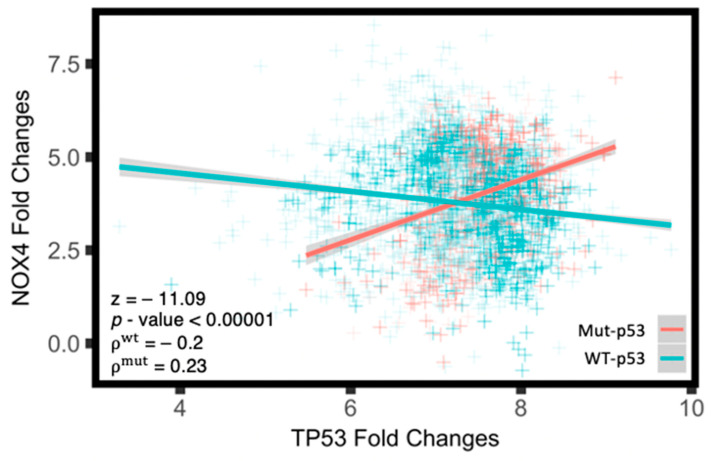
Mut-p53 mRNA positively correlate with NOX4 mRNA in the Pan-Cancer cohort. The Preliminary Pan-Cancer cohort was divided into patients with WT-*TP53* or Mut-*TP53*, and a scatterplot was generated to visualize the relative mRNA (RSEM) values of p53 and NOX4. Linear regression was used to assess for broad correlations followed by Spearman’s test to calculate the *ρ* values, and two-tail Fisher’s r-to-z transformation to assess for the significance in *ρ* value difference. *n*^WT-p53^ = 2368, *n*^mut-p53^ = 888.

In the Preliminary Pan-Cancer Dataset, we sorted tumor samples as having WT-*TP53* or any of the 15 most common hot-spot mutants (Mut-p53) and found that in tumors with Mut-*TP53*, the level of p53 mRNA positively correlates with NOX4 mRNA (*ρ* = 0.23), whereas WT-*TP53* mRNA negatively correlates with NOX4 mRNA (*ρ* = −0.2, Figure 3). This corroborates our earlier findings, where WT-p53 suppresses whereas Mut-p53 enhances NOX4 expression in established epithelial tumor cell culture models [16,17]. This also suggests that *TP53* mutation status may play a critical role in cellular processes previously shown to involve NOX4, such as EMT, cell migration, angiogenesis and other genetic and metabolic programs that promote cancer progression [15,16,20,37,38].

### 3.4. NOX4 Shows Strong Positive Correlations with Transcriptional Programs of Cancer Progression Including EMT, Cell Migration, Cell Adhesion, and Angiogenesis

**Figure 4:**
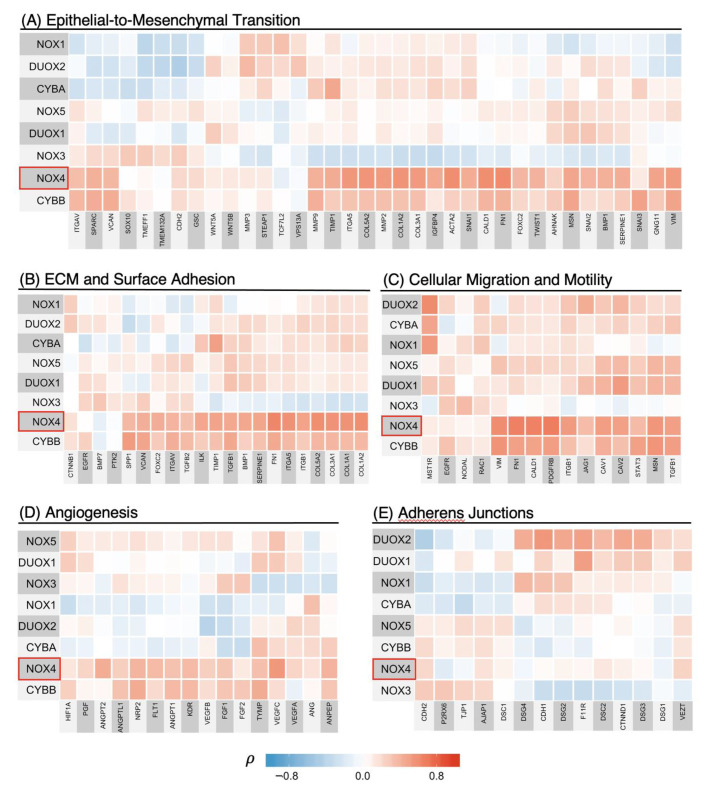
NOX4 positively correlates with several genetic programs of cancer progression in the Pan-Cancer cohort. Spearman’s rank correlation coefficients, *ρ*, were calculated and mapped to assess the relationships between individual NADPH oxidases and various genetic programs of cancer progression, such as epithelial-to-mesenchymal transition (EMT), extracellular matrix (ECM)/surface adhesion, cell migration and motility and angiogenesis ((**A**–**D**), respectively) using the Final Pan-Cancer Dataset. *NOX4* expression shows strong positive correlations with genes in programs (**A**–**D**), but poor correlations with epithelial marker genes in (**E**). The direction of correlation is indicated by the color: blue is a negative whereas red is a positive correlation, and the strength of the correlation is represented by the color intensity. Genes listed with each program and their corresponding functions in cancer are provided in Table S1.

To our knowledge, no other study has explored the involvement of the individual NADPH oxidase family of enzymes in the context of the genetic programs of cancer progression in a Pan-Cancer cohort. Therefore, we calculated correlation matrices of each of the NADPH oxidase family enzymes with genes previously described as involved in EMT, cellular adhesion and ECM, migration and motility, and angiogenesis using the Final Pan-Cancer Dataset (Figure 4). Each square on the heatmaps represents the Spearman’s *ρ*-value of the corresponding genes on the *x*- and *y*-axis. The *ρ*-value indicates the presence of a monotonic relationship and is insensitive to outliers. Here, the possible range of the *ρ*-value of −1 to +1 is mapped to the blue–white–red color gradient. Lastly, unsupervised clustering was performed on each matrix to discern broad relationships.

Here, we report that NOX4 is not only involved in the EMT genetic program, but also in transcriptional programs promoting ECM production, cellular adhesion, migration, motility, and angiogenesis (Figure 4A–D).

In the EMT genetic program, *NOX4* expression is strongly associated with several canonical mesenchymal markers such as vimentin (VIM) and fibronectin (FN1) (*ρ* = 0.5, *ρ* = 0.6, respectively, Figure 4A). NOX4 is also linked to genetic markers known to promote extracellular matrix assembly, cell surface adhesion, migration and motility (Figure 4B,C). Such strong correlations with genes involved in migration suggest NOX4 is a key player in cancer cell dissemination. Several canonical factors that promote endothelial cell growth and blood vessel development also correlate with NOX4 mRNA levels (Figure 4D). Of note, vascular endothelial growth factor C (VEGFC), neuropilin-2 (NRP2), vascular endothelial growth factor receptor 2 (KDR), and angiopoietin 2 (ANGPT2)—all of which promote angiogenesis and vascularization—are closely linked to NOX4 expression (*ρ* = 0.55, 0.40, 0.37, 0.42, respectively). In contrast, NOX4 is not strongly associated with cell-to-cell adhesion molecules characteristic of the epithelial phenotype (Figure 4E). Together, these data suggest that NOX4 expression is associated with aggressive cancer phenotypes, such as enhanced cell migration and dissemination, but not in regaining the epithelial phenotype.

Interestingly, NOX2 (encoded by *CYBB*) seems to have similar, although less striking, correlation patterns to NOX4 with all the cancer progression-related markers we surveyed (Figure 4A–D). Although NOX2 appears to be a weaker indicator of EMT progression, these similarities prompted us to further investigate the source of NOX2 and how it may be associated with these programs.

### 3.5. NOX2 (CYBB) Is Tightly Linked to Other Macrophage Markers

The involvement of *CYBB* (NOX2) in the EMT genetic program has not been well documented. However, NOX2 is known to be expressed in macrophages and is the major source of macrophage-derived ROS [39]. In addition, the presence of tumor-associated macrophages (TAMs) in the tumor microenvironment has been shown to promote cancer cell proliferation, EMT, and angiogenesis [40,41]. Therefore, we hypothesized that the correlation between NOX2 and cancer-progression-related events could be attributed, in large part, to macrophage infiltration.

**Figure 5:**
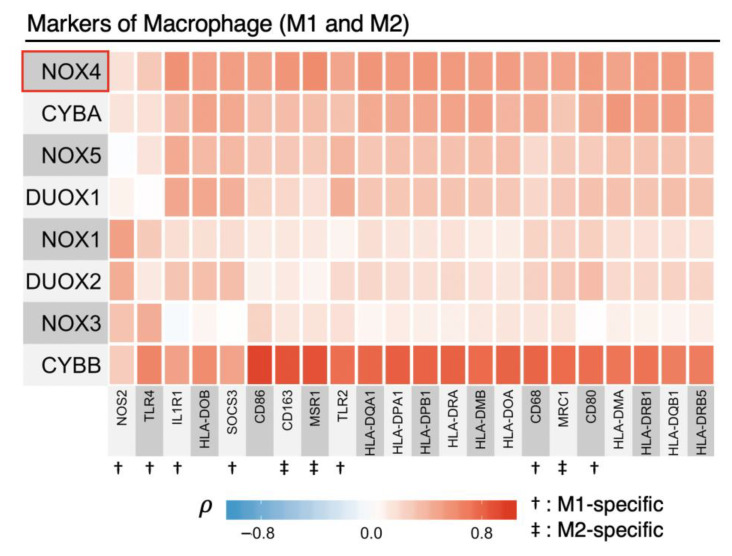
The source of CYBB (NOX2) is likely due to tumor-associated macrophages (TAMs). Spearman’s rank correlation coefficients, *ρ*, were calculated in the Final Pan-Cancer Dataset to assess for monotonic relationships between the individual NADPH oxidases and canonical markers of M1 and M2 macrophages. *CYBB* (*NOX2*) expression strongly correlates with most markers of M1 and M2 classification of macrophages. A key to gene abbreviations listed is provided in Table S2.

While the canonical definitions of macrophage polarization to ‘M1/M2′ subtypes do not reflect the range of biological functions that TAMs can engage, we curated a panel of macrophage markers that includes both M1 and M2 subtypes. We discovered a striking association between NOX2 and macrophage signatures that strongly suggests that the major source of NOX2 is derived from TAMs (Figure 5). Although NOX2 is considered the primary source of ROS in macrophages, recent studies revealed that other less abundant NOX enzymes can affect the fate and function of macrophages, including DUOX1, NOX1 and NOX4 [42,43,44]. For example, studies with Nox1/Nox2 double-knockout mice suggest both oxidases support monocyte differentiation into macrophages [43]. Furthermore, observations with Nox4 knock-out mice indicate the absence of Nox4 results in several proinflammatory phenotypes, including increased macrophage Nox2 expression and enhanced infiltration of proinflammatory macrophages in tumors of an induced fibrosarcoma model [44]. Taken together, we must consider that TAM and TAM-derived NOX2 are linked to the aforementioned genetic programs of cancer progression, and that this may, in part, involve a direct interplay between Nox2 and Nox4 within TAMs.

### 3.6. TP53 Mutations Alter NOX4 mRNA Correlation with EMT Gene Expression

**Figure 6:**
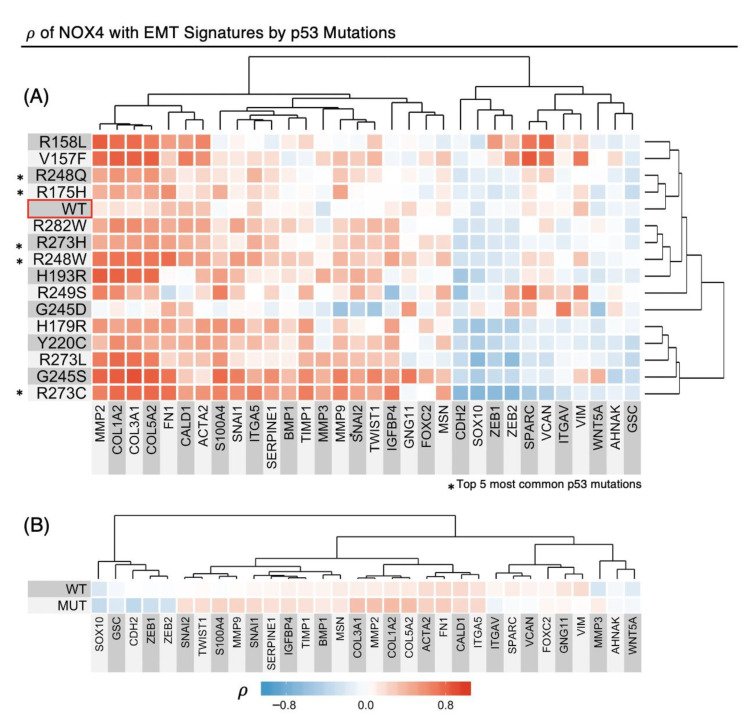
Common *TP53* mutations strengthen the correlation coefficients of *NOX4* mRNA with EMT genes. (**A**) Samples in the Final Pan-Cancer Dataset were identified as harboring WT-*TP53* or any of the indicated common Mut-*TP53*, and the Spearman’s rank correlation coefficients *ρ* were calculated for *NOX4* with the EMT genes indicated on the *x*-axis. (**B**) All p53 mutations studied were collapsed into one group, indicated by mutant (MUT), versus wild-type (WT), and the resulting *ρ* of *NOX4*-EMT genes were mapped. Unsupervised clustering was performed for both (**A**) and (**B**).

Since NOX4 is the strongest correlate of genetic programs of cancer progression, we explored factors that could further enhance or alter this association. Previously, we showed that WT and several p53 mutants can alter both TGF-β-dependent and -independent NOX4 expression [16,17]. Therefore, we sorted the Final Pan-Cancer cohort by many common p53 mutations and determined the *ρ*-values of the indicated EMT signature in relation to NOX4 mRNA levels (Figure 6A). Here, each square represents the *ρ*-value of NOX4 to the gene on the x-axis, and each row represents patients with different *TP53* mutation statuses indicated on the y-axis. Many p53 mutants, such as p53-V157F, R158L, R273L, H193R, G245S, R248W, R273C, R273H, and R248Q, appear to augment the correlation between NOX4 with key EMT markers such as collagens (COL1A2, COL3A1, COL5A2), metalloproteases (MMP2, MMP9) and fibronectin (FN1). Further, when we collapsed the p53 mutants we studied into a single group, the increases in *ρ* of NOX4 and several EMT genes were still apparent, e.g., collagens, MMP2/9, and SNAI1/2 transcription factors (Figure 6B).

### 3.7. NOX4 Expression Affects Survival Outcome Depending on p53 Status

**Figure 7:**
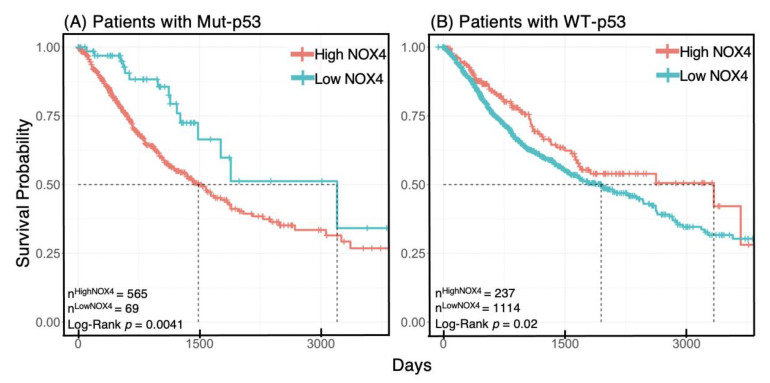
Increased NOX4 mRNA is deleterious to survival in the presence of mut-p53. Patients in the Final Pan-Cancer Dataset were identified as harboring a mutated form of *TP53* (**A**) or WT-*TP53* (**B**). Optimized cut-off points were then calculated for high vs. low NOX4 mRNA levels for each group and Kaplan–Meier plots and Log-rank tests were used to estimate survival probability and *p*-values, respectively. Ten-year survival (3650 days) is shown in both (**A**) and (**B**).

So far, we have shown the involvement of NOX4 in cancer progression and how *TP53* mutations affect *NOX4* expression and its correlation with key EMT signatures, but how these factors influence clinical parameters is unknown. Therefore, we explored whether NOX4 in association with *TP53* mutation status plays a role in patient survival. We classified patients’ tumors as either having WT-*TP53* or Mut-*TP53* and estimated the patient survival probability in relation to NOX4 levels using Kaplan–Meier plots and calculated the *p*-value using the non-parametric Log-Rank tests. We found opposing survival likelihood based on p53 mutation status in patients—higher NOX4 levels are associated with significantly better ten-year survival in patients with WT-*TP53*, but worse in patients with Mut-*TP53* (Figure 7). The median survival of patients with WT-*TP53* and relatively low NOX4 is about six years and about eight years with high NOX4. In contrast, in patients with Mut-*TP53*, the median survival is reversed—about four years longer with low NOX4 expression. This finding suggests that Mut-p53 augments NOX4-related malignant processes, but the means by which NOX4 and WT-p53 could be protective remain unclear.

### 3.8. Mut-p53 and WT-p53 Divergently Affect NOX4 Correlations with Cell Proliferation and Apoptosis

**Figure 8:**
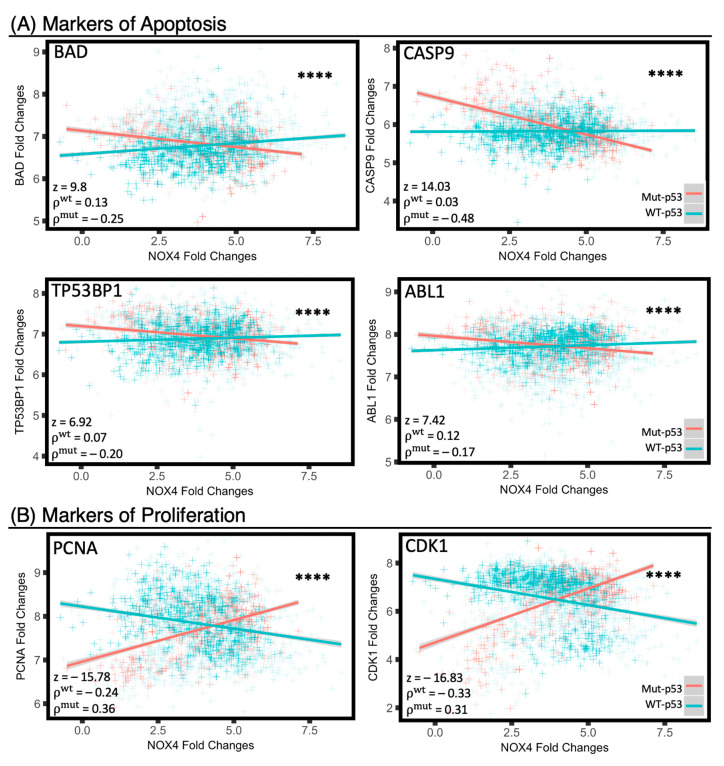
Mut-p53 and WT-p53 divergently affect NOX4 correlations with cell proliferation and apoptosis markers. From the Preliminary Pan-Cancer Dataset, samples were identified as either harboring WT-*TP53* or Mut-*TP53*. Scatterplots were generated to visualize relationships between NOX4 and canonical markers of cell apoptosis (**A**) and cell proliferation (**B**). Spearman’s rank correlation coefficients were calculated and compared between WT and Mut-*TP53* group using two-tail Fisher’s r-to-z transformation. *n*^wt^ = 2368, *n*^mut^ = 888. **** *p*-value < 1 × 10^−5^. Genes associated with apoptosis or proliferation and their corresponding functions are provided in Table S3.

To explore how NOX4 is influenced by WT-p53 or Mut-p53 to produce favorable or deleterious survival outcomes, respectively, we evaluated whether NOX4 is associated with other clinically relevant cellular processes such as cell proliferation and apoptosis. Previously, NOX4 has been implicated in these cellular processes in several cancer models but not in a Pan-Cancer cohort in relation to p53 [14,21,22,23]. We employed linear regression to assess for broad relationships, followed by Spearman’s rank test and comparison of *ρ*-values using the Fisher r-to-z transformation. In tumor samples with Mut-*TP53*, *NOX4* overexpression is positively correlated with proliferation markers such as proliferating cell nuclear antigen (PCNA) and cyclin-dependent kinase 1 (CDK1, Figure 8A), and negatively correlated with apoptosis-related markers such as Bcl-associated agonist of cell death (BAD), caspase 9 (CASP9), tyrosine-protein kinase ABL1 (ABL1), and TP53-binding protein 1 (TP53BP1) (Figure 8B). In contrast, the opposite trends were observed in tumor samples with WT-*TP53:* NOX4 is negatively correlated with the proliferation marker PCNA and CDK1 in tumors with WT-*TP53*.

## 4. Discussion

Previously, our lab has demonstrated that TGF-β-induced NOX4-dependent EMT is modulated by *TP53* status; WT-p53 suppresses whereas several mutant forms of p53 augment *NOX4* expression and NOX4-dependent cell migration in vitro [16,17]. Expression of dominant-negative NOX4 or *NOX4*-specific shRNAs reduced TGF-β-induced EMT events and wound closure [11,16,17]. This occurs primarily through epigenetic mechanisms, since overexpression of p300, a transcriptional co-regulator and histone acetyltransferase (HAT), enhanced mut-p53-mediated NOX4 induction, whereas HAT-inactive p300 reduced *NOX4* expression [17]. Moreover, we found histone deacetylase (HDAC) inhibitors could relieve the WT-p53-mediated repression of NOX4.

In support of our early in vitro findings that NOX4 is involved in cancer progression and migration, Zhang et al., showed *Nox4* silencing by shRNA decreased the TGF-β-induced migration of 4T1 murine breast cancer cells and significantly attenuated 4T1 cells metastasis to the lungs and bones of nude mice [15]. In another study, the Oncomine database was utilized to retrieve six different studies showing *NOX4* is highly expressed in colorectal carcinoma [19]. Statistical and immunohistochemical analyses showed increases in *NOX4* expression when compared to adjacent non-malignant tissue [13,18,19]. While these studies suggest NOX4 is involved in cancer progression, they did not address the relationship between NOX4 and *TP53* mutations. Our preliminary analysis of data in TCGA indicated that *NOX4* transcript levels are elevated in human primary breast, pancreatic, and head and neck tumors detected with several common *TP53* hotspot mutations [17].

With the conclusion of The Cancer Genome Atlas (TCGA) data curation, we have a unique opportunity to explore the roles of NOX4 and p53 in thousands of primary tumor samples where we used an unbiased, Pan-Cancer approach. This method allowed us to increase the statistical power dramatically. The Pan-Cancer approach is a means to illuminate the possible molecular mechanisms that are applicable regardless of tissue of origin. We first found that *NOX4* expression directly corresponds to cancer progression in several tumor types: bladder urothelial carcinoma, thyroid carcinoma and esophageal carcinoma (Figure 2A–C). However, we found that in breast invasive carcinoma, *NOX4* mRNA levels remain unchanged in the different pathological stages (Figure 2D). Nonetheless, in light of recent work showing that tissue origin does not completely dictate the molecular pattern of the tumor [24], we combined all samples from all available tissue sources and formed a Pan-Cancer cohort with several thousand samples as a means to identify roles of NOX4 and p53 in the genetic programming across cancer types. Consistent with our previous work [16,17], we found that WT-*TP53* mRNA is inversely correlated with *NOX4* mRNA, whereas Mut-*TP53* mRNA is positively correlated with *NOX4* expression in the Pan-Cancer cohort (Figure 3). By extension, this suggests that WT- and Mut-p53 differentially modulate NOX4-dependent mechanisms and cellular events.

We then examined whether NOX4 and other enzymes in the NADPH oxidase family are broadly associated with EMT and cancer-related genetic programs and found that only NOX4 is strongly linked to key genetic programs of cancer progression, including EMT, extracellular matrix production and cell surface adhesion, cellular migration and motility, and angiogenesis (Figure 4). Each gene-set was curated using GSEA and the current literature and is more extensive than the canonical gene-lists associated with each cellular function. Therefore, we did not expect any specific proteins to tightly correlate with all the genes listed in each genetic program. Still, the vast majority of genes in each genetic program of cancer progression positively correlates with *NOX4* expression, therefore suggesting the involvement of NOX4 in these cellular functions. This important implication prompted us to further investigate factors that may modulate the effects of NOX4 in the tumor environment.

We uncovered that most common *TP53* hotspot mutations generally strengthen the correlation of *NOX4* with the EMT genetic program, and this effect was still seen when gene expression profiles from all tumors with *TP53* mutations were merged together and compared as a group vs. WT-*TP53* tumors (Figure 6). More importantly, *TP53* mutation status alters survival outcome based on *NOX4* expression: increased *NOX4* expression is protective in patients with WT-*TP53* tumors but deleterious in patients with Mut-*TP53* (Figure 7). Together, this suggests the mechanisms and targets of NOX4-generated ROS are dependent on *TP53* mutation status.

**Figure 9:**
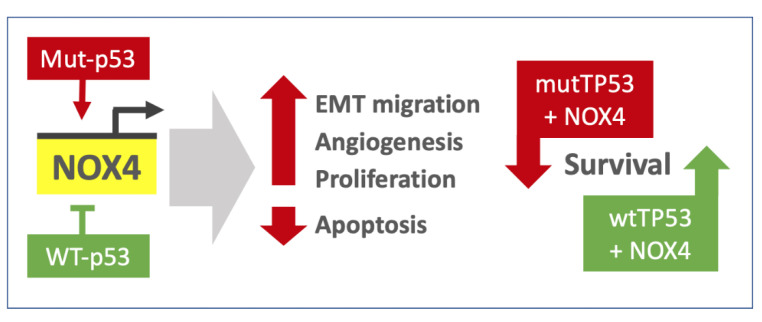
Schematic summary. Divergent effects of mutant versus WT p53 on *NOX4* expression and downstream consequences on cancer progression programs (**left**). Contrasting effects of high *NOX4* expression on survival outcomes in patients with WT versus mutant *TP53* tumors (**right**).

To further investigate the mechanisms by which WT- and Mut-p53 may affect NOX4 to produce divergent survival outcomes and further increase the statistical power of this analysis, we examined the Preliminary Pan-Cancer dataset from over 3200 samples. Here, we found that in patients with Mut-*TP53*, increases in *NOX4* expression are directly associated with decreases in key apoptotic markers and increases in proliferation markers, whereas the opposite trends were found in patients with WT-*TP53* (Figure 8). This is the first time, to our knowledge, that NOX4 is shown to exhibit divergent effects on apoptosis and proliferation in a Primary Pan-Cancer cohort. Together with our previous in vitro mechanistic data showing p53 modulates *NOX4* expression epigenetically through histone acetylation [17], we present novel evidence supporting the role of NOX4 in cancer progression and demonstrate important implications of *NOX4* expression in patient survival in a wide-ranging Pan-Cancer analysis. The effects of NOX4 are altered under the influence of WT versus Mut-p53, suggesting mutations in *TP53* switch downstream signaling targets to enhance cancer cell dissemination and proliferation via NOX4-generated ROS (Figure 9). In contrast, WT-p53 is known as ‘the guardian of the genome’ since it arrests cell proliferation and promotes apoptosis in response to DNA damage. Recent work by Helfinger et al. [45], suggests that the redox tone established by Nox4 in mice has a role in promoting higher WT-*TP53* mRNA and protein production and maintaining genomic stability by participating in the DNA damage response; interestingly, these studies showed Nox4-deficient mice are more susceptible to tumor formation in models of inflammation and chemical-induced carcinogenesis. Thus, the issues of whether NOX4 and the ROS it generates suppress tumorigenesis or promote cancer progression should be considered in the context of *TP53* mutation status, since our analysis of thousands of primary human tumor samples suggests that both functions of NOX4 are plausible.

Although the statistical power is high with our Pan-Cancer method, there are several limitations in this broad survey approach. First, since TCGA is a repository of primary tumor data that provide a “snapshot” of each tumor’s molecular profile at the time of harvest, these data cannot be used to infer causation. Second, because of the unequal prevalence of different types of cancer and varying quality of specimens, the 23 cancer types were not equally represented in the Pan-Cancer cohorts. Finally, in using the TCGA repository, we are unable to define the precise cellular sources of specific mRNAs because the tumor samples are a heterogeneous mixture that is not sorted by cell type. Thus, many cell types including cancer-associated fibroblasts, vascular cells, macrophages and other inflammatory cells that cross-talk with transformed cells in the tumor microenvironment are potential contributors to the gene expression profiles that promote cancer progression. For example, two recent reports noted relatively higher stromal NOX4 expression in myofibroblasts and fibroblasts adjacent to cancer cells in several types of solid tumors and suggested therapies targeting stromal NOX4 could reduce tumor growth [46,47]. Later studies using NOX4 silencing or inhibition approaches suggested that fibroblastic NOX4 could limit the accessibility of CD8^+^ T-cells to the intratumoral microenvironment [48]. We also showed high tumor NOX2 (*CYBB*) expression that was tightly correlated with canonical M1 and M2 markers, and likely reflects TAMs, is also closely associated with many of the same cancer promoting programs linked to NOX4. Thus, future work should examine, in detail, the relevant cell types within the tumor microenvironment that could synergize to promote or suppress cancer progression programs identified in our Pan-Cancer analysis. Despite the limitations, our Pan-Cancer studies describe novel findings involving NOX4 and p53 signaling in the cancer environment with translational potential for cancer diagnostics and therapeutics.

## 5. Conclusions

In summary, our Pan-Cancer analysis of human tumor data indicates NOX4 can contribute to several gene expression programs that promote cancer progression in many tumor types with common *TP53* mutations, and that *TP53* mutations could “switch” NOX4 from being protective and an indicator of good prognosis to deleterious outcomes of cancer progression. Therapeutic strategies targeting NOX4 or the ROS it generates should take into consideration *TP53* mutation status, as NOX4 may provide patient survival benefits in the presence of WT-p53 but may represent a worthwhile target in advanced cancers with mutated p53.

## Figures and Tables

**Table 1:** Patient characteristics of the Preliminary Pan-Cancer Dataset and Final Pan-Cancer Dataset.

Study	Abbrv.	Prelim. *n*	Final *n*
Bladder Urothelial Carcinoma	BLC A	309	294
Brain Lower Grade Glioma	LGG	299	247
Breast invasive carcinoma	BRCA	246	172
Cervical squamous cell carcinoma and endocervical adenocarcinoma	CESC	113	113
Colon adenocarcinoma	COAD	562	39
Esophageal carcinoma	ESAD	48	50
Glioblastoma multiforme	GBM	43	27
Head and Neck squamous cell carcinoma	HNSC	102	69
Kidney renal clear cell carcinoma	KIRC	86	86
Kidney renal papillary cell carcinoma	KIRP	10	10
Liver hepatocellular carcinoma	LIHC	29	29
Lung adenocarcinoma	LUAD	171	153
Lung squamous cell carcinoma	LUSC	173	137
Mesothelioma	MESO	87	87
Ovarian serous cystadenocarcinoma	OV	194	158
Pancreatic adenocarcinoma	PAAD	59	59
Prostate adenocarcinoma	PRAD	21	18
Rectum adenocarcinoma	READ	192	17
Sarcoma	SARC	27	27
Stomach adenocarcinoma	STAD	86	54
Thyroid carcinoma	THCA	212	106
Uterine Carcinosarcoma	UCS	17	17
Uterine Corpus Endometrial Carcinoma	UCEC	170	25
Total		3256	1994

## Data Availability

The datasets supporting the conclusions of this article are freely available at TCGA: http://cancergenome.nih.gov, Broad GDAC Firehose: https://gdac.broadinstitute.org/ (doi:10.7908/C1K9371X), and cBioPortal: http://www.cbioportal.org/ [22,23]. Codes used in the analyses are available at Github: https://github.com/wfma/Ma_2018 (doi:10.5281/zenodo.1249667).

## Supplementary Materials

The following information on genes analyzed in this study available online at https://www.mdpi.com/2076-3921/10/2/235/s1: Table S1: Genes associated with EMT, ECM, migration, angiogenesis and cellular junctions, and their functions in cancer (for Figure 4); Table S2. Markers of M1 and M2 macrophages (for Figure 5); Table S3: Genes associated with apoptosis and proliferation (for Figure 8).
