# Supplementary material for: Pan-Cancer Analysis Shows TP53 Mutations Modulate the Association of NOX4 with Genetic Programs of Cancer Progression and Clinical Outcome
Source: Antioxidants (Basel). 2021 Feb 4;10(2):235. doi: 10.3390/antiox10020235 (PMC7915715; doi:10.3390/antiox10020235)
Supplement: Supplementary file 1 [file antioxidants-10-00235-s001.pdf]

Supplementary Files

Table S1. Genes associated with EMT, ECM, migration, angiogenesis and cellular junctions and their functions in cancer (for Fig. 4).

(A) Epithelial-to-Mesenchymal Transition

Gene Name	Abbreviation	Ref.	Function and Characteristics
Alpha-smooth muscle actin	ACTA2	[1, 2]	Required for lung cancer metastasis and progression of other carcinomas.
Neucleoprotein AHNAK	AHNAK	[3, 4]	Upregulated in partial-EMT or mesenchymal cells. Knockdown of AHNAK reduces cell migration in mesothelioma cell lines.
Bone morphogenic protein 1	BMP1	[5]	Involved in matrix assembly.
Caldesmon 1	CALD1	[4, 6]	Overexpressed in mesenchymal cells. Calcium sensitive, regulates smooth muscle contraction.
N-Cadherin	CDH2	[7]	Activated in EMT via SNAIL or bHLH pathway and overexpression is a hallmark of EMT. The cadherin switch from E to N triggers loss of epithelial cell association.
Collagen 1 A2	COL1A2	[4, 7]	Driven by SNAIL, activated in EMT.
Collagen 3 A1	COL3A1	[7]	Driven by SNAIL, activated in EMT.
Collagen 5 A2	COL5A2	[7]	Driven by SNAIL, activated in EMT.
Fibronectin	FN1	[6, 7]	Activated in EMT via SNAIL or bHLH pathway. A part of the extracellular matrix. Canonical indicator of EMT.
Forkhead box protein C2	FOXC2	[8]	Indirect repressor of E-cadherin, which is an epithelial marker.
G protein subunit gamma 11	GNG11	[9]	Maybe an indicator of EMT. Differentially expressed in EMT meta-analysis.
Insulin like growth factor binding protein 4	IGFBP4	[10]	Overexpression leads to tumor growth and EMT phenotype in renal cell carcinoma.
Integrin alpha subunit 5	ITGA5	[11]	May promote tumor invasiveness and cell migration.
Integrin alpha V	ITGAV	[11]	Receptor for ECM, i.e. FN1, VTN, and MMP.
Matrix metalloprotease 2	MMP2	[7]	Driven by SNAIL, activated in EMT.
Matrix metalloprotease 3	MMP3	[7]	Driven by SNAIL, activated in EMT.
Matrix metalloprotease 9	MMP9	[7]	Driven by SNAIL, activated in EMT.
Moesin	MSN	[7]	Driven by SNAIL, activated in EMT. Likely to be involved in connecting cytoskeletal

			structures to the plasma membrane. Plays a role in proliferation, migration and adhesion of lymphoid cells.
Plasminogen activator inhibitor 1	SERPINE1	[6, 7]	Driven by SNAIL, activated in EMT. Involved in extracellular matrix organization.
Zinc finger protein 1	SNAI1	[7]	Down regulates epithelial markers and upregulates mesenchymal markers.
Zinc finger protein 2	SNAI2	[7]	Down regulates epithelial markers and upregulates mesenchymal markers.
Zinc finger protein 3	SNAI3	[7]	Also in the zinc finger protein family, but exact role in EMT is unclear.
Transcription factor SOX10	SOX10	[12]	Regulates mesenchymal cell attributes. Drives EMT and contributes to stem/progenitor activities in mammary cells.
Secreted protein acidic and rich in cystine	SPARC	[7]	Activated in EMT via SNAIL or bHLH. Involved in the response to growth factors and extracellular matrix organization.
Six Transmembrane Epithelial Antigen Of The Prostate 1	STEAP1	[13, 14]	May play a role in EMT, often up-regulated in several cancer cell lines.
T-cell factor 7L2	TCF7L2	[15]	May play a role in EMT and may be influenced by SNAIL regulation.
Tissue inhibitor of metalloproteinase-1	TIMP1	[16]	Overexpression can induce EMT phenotypes via TWIST1.
transmembrane protein with EGF like and two follistatin like domains 1	TMEFF1	[14]	May suppress cancer growth but also may be a mesenchymal marker.
transmembrane protein 132A	TMEM132A	[8, 14]	May play a role in EMT.
Versican core protein	VCAN	[6]	Play a role in connecting cells to ECM, can also regulate motility growth and differentiation.
Twist-related protein 1	TWIST1	[9]	Indirect repressor of E-cadherin, which is an epithelial phenotype.
Vimentin	VIM	[9, 17, 18]	Expression can induce EMT and is a poor prognostic marker.
Vacuolar Protein Sorting 13 Homolog A	VSP13A	[16]	May play a role in EMT.
Protein WNT-5A	WNT5A	[19]	Can act via non-canonical pathways to promote tumor cell invasion.

(B) ECM and Surface Adhesion

**Gene Name**	**Abbreviation**	**Ref.**	**Function and Characteristics**

Bone morphogenic protein 1	BMP1	[5]	Involved in matrix assembly.
Bone morphogenic protein 7	BMP7	[5]	Promotes EMT in gastric and prostate cancer. May have paradoxical effects in different cancer types.
Collagen 1 A1	COL1A1	[4, 7]	Driven by SNAIL, activated in EMT. A normal part of the extracellular matrix.
Collagen 1 A2	COL1A2	[4, 7]	Driven by SNAIL, activated in EMT. A normal part of the extracellular matrix.
Collagen 3 A1	COL3A1	[7]	Driven by SNAIL, activated in EMT. A normal part of the extracellular matrix.
Collagen 5 A2	COL5A2	[7]	Driven by SNAIL, activated in EMT. A normal part of the extracellular matrix.
Catenin beta	CTNNB1	[6]	A part of the adherens junction as a component of the E-Cadherin-Catenin complex.
Epidermal growth factor	EGFR	[6, 20]	Increases MUC1 interaction with CTNNB1. E-cadherin-mediated cell-to-cell contact can induce EGFR.
Fibronectin	FN1	[6, 7]	Activated in EMT via SNAIL or bHLH pathway. A part of the extracellular matrix. Canonical indicator of EMT.
Forkhead box protein C2	FOXC2	[8]	Indirect repressor of E-cadherin, which is an epithelial phenotype.
Integrin linked protein kinase	ILK	[6]	Focal adhesion protein mediates cell architecture, adhesion and anchorage-dependent growth.
Integrin alpha subunit 5	ITGA5	[11]	May promote tumor invasiveness and cell migration.
Integrin alpha V	ITGAV	[11]	Receptor for ECM, i.e. FN1, VTN, and MMP.
Integrin beta 1	ITGB1	[6, 21]	Core regulator of integrin-linked kinase, focal adhesion kinases and others involved in cellular adhesion. Promotes endothelial cell motility and angiogenesis.
Focal adhesion kinase 1	PTK2	[6]	Critical in regulating cell migration adhesion an spreading.
Plasminogen activator inhibitor 1	SERPINE1	[6, 7]	Driven by SNAIL, activated in EMT. Involved in extracellular matrix organization.
Osteopontin	SPP1	[22]	Overexpression leads to cancer cell migration
Transforming growth factor beta 1	TGFB1	[23, 24]	Can induce EMT and migration of cancer cells.
Transforming growth factor beta 2	TGFB2	[6,14]	Can induce EMT and migration of cancer cells.
Tissue inhibitor of metalloproteinase-1	TIMP1	[16]	Overexpression can induce EMT phenotypes via TWIST1.

Versican core protein	VCAN	[6]	Play a role in connecting cells to ECM, can also regulate motility growth and differentiation.

(C) Cellular Migration and Motility

**Gene Name**	**Abbreviation**	**Ref.**	**Function and Characteristics**
Caldesmon 1	CALD1	[4, 6]	Overexpressed in mesenchymal cells. Calcium sensitive, regulates smooth muscle contraction.
Caveolin 1	CAV1	[25]	Biphasic effect where CAV1 is tumor-suppressing in the early stages but tumor-promoting in the later stages of cancer.
Caveolin 2	CAV2	[26]	Silencing CAV2 reduces cell proliferation, migration, and invasion in renal cell carcinoma.
Epidermal growth factor	EGFR	[6, 20]	Increases MUC1 interaction with CTNNB1. E-cadherin-mediated cell-to-cell contact can induce EGFR.
Fibronectin	FN1	[6, 8]	Activated in EMT via SNAIL or bHLH pathway. A part of the extracellular matrix. Canonical indicator of EMT.
Integrin beta 1	ITGB1	[6, 21]	Core regulator of integrin-linked kinase, focal adhesion kinases and others involved in cellular adhesion. Promotes endothelial cell motility and angiogenesis.
Protein jagged 1	JAG1	[6]	Enhances fibroblast growth factor-induced angiogenesis.
Moesin	MSN	[8]	Driven by SNAIL, activated in EMT. Involved in connecting cytoskeletal structures to the plasma membrane. Plays a role in proliferation, migration and adhesion of lymphoid cells.
Macrophage stimulating protein receptor	MST1R	[6]	Signal can promote epithelial cell migration, proliferation and survival.
Nodal homolog	NODAL	[6]	May positively regulate angiogenesis and cell-cell adhesion. Critical in embryonic development of the mesoderm.
Platelet-derived growth factor receptor beta	PDGFRB	[6]	Essential role in embryonic development, cell proliferation and survival, differentiation, chemotaxis, migration, blood vessel formation, etc.
Ras-related c3 botulinum toxin substrate 1	RAC1	[6]	Small GTPase involved in many cellular responses including epithelial cell polarization and growth-factor dependent membrane ruffles. Mediates cell migration and adhesion assembly.

Signal transducer and activator of transcription 3	STAT3	[6]	Mediates cellular responses in response to stimuli. May positively regulate angiogenesis and growth factor-dependent cell proliferation.
Vimentin	VIM	[8, 17, 18]	Expression can induce EMT and is a poor prognostic marker.

(D) Angiogenesis

Gene Name	Abbreviation	Ref.	Function and Characteristics
Angiotensinogen	ANG	[6]	Apart from regulating blood pressure, it can positively regulate endothelial cell migration,
Angiopoietin 1	ANGPT1	[6]	Competes and modulates ANGPT2. Regulates angiogenesis and endothelial cell survival. In migrating endothelial cells, it can induce focal adhesion complexes and stimulate angiogenesis/blood vessel maturity.
Angiopoietin 2	ANGPT2	[6]	In concert with VEGF facilitates endothelial cell migration and services as a permissive signal for angiogenesis.
Angiopoietin related protein 1	ANGPTL1	[14]	Specific for vascular endothelium and highly expressed in vascular tissue.
Aminopeptidase N	ANPEP	[6]	Processes angiotensin.
Fibroblast growth factor 1	FGF1	[6]	It has an important role in regulating cell survival, division, angiogenesis, cell differentiation and cell migration. It can induce angiogenesis.
Fibroblast growth factor 2	FGF2	[6]	Potent mitogen. Can induces angiogenesis.
Vascular endothelial growth factor receptor	FLT1	[6]	May limit VEGFA binding. Regulates angiogenesis. Can promote endothelial cell proliferation, survival, and angiogenesis in adulthood (as opposed to embryonic development).
Hypoxia inducible factor 1 alpha	HIF1A	[6]	Master regulator of hypoxia response. In hypoxic environment of tumor, it can induce angiogenesis.
Vascular endothelial growth factor receptor 2	KDR	[6]	Receptor for VEGFA, VEGFC, and VEGFD. Essential regulator of angiogenesis.
Neuropilin-2	NRP2	[6]	Positive regulator of endothelial cell migration and proliferation.
Placenta growth factor	PGF	[6]	Active in angiogenesis and simulates endothelial cell proliferation and migration.
Thymidine phosphorylase	TYMP	[6]	Maintains integrity of blood vessels and promotes endothelial cell growth and angiogenesis.

Vascular endothelial growth factor A	VEGFA	[6]	Growth factor that induces angiogenesis, vasculogenesis and endothelial cell growth.
Vascular endothelial growth factor B	VEGFB	[6]	Growth factor for endothelial cells.
Vascular endothelial growth factor C	VEGFC	[6]	Growth factor that induces angiogenesis, vasculogenesis and endothelial cell growth.

(E) Adherens Junctions

**Gene Name**	**Abbreviation**	**Ref.**	**Function and Characteristics**
Adherens junction-associated protein1	AJAP1	[14]	Plays a role in cell adhesion and cell migration.
E-cadherin/ Cadherin-1	CDH1	[6, 27, 28]	Regulates cell-cell adhesions, loss during EMT, a marker of epithelial phenotype.
N-Cadherin Cadherin-2	CDH2	[14]	Calcium-dependent cell adhesion protein. Overexpression of N-cadherin is a hallmark of EMT.
Catenin delta	CTNND1	[6]	Regulates cell adhesion properties of C,E, and N-cadherins
Desmocollin	DSC1	[14]	Found primarily in epithelial cells where they constitute the desmosome cell-cell junction and are required for cell adhesion and desmosome formation.
Desmocollin-2	DSC2	[6]	Major component of the desmosome, which is an important cell-to-cell adhesion molecule.
Desmoglein 1	DSG1	[14]	Member of the desmoglein protein subfamily, which, along with desmocollins, are cadherin-like transmembrane glycoproteins that are major constituents of the desmosome.
Desmoglein 2	DSG2	[14]	Member of the desmoglein protein subfamily, which, along with desmocollins, are cadherin-like transmembrane glycoproteins that are major constituents of the desmosome.
Desmoglein 3	DSG3	[14]	Member of the desmoglein protein subfamily, which, along with desmocollins, are cadherin-like transmembrane glycoproteins that are major constituents of the desmosome
Desmoglein 4	DSG4	[14]	Member of the desmoglein protein subfamily, which, along with desmocollins, are cadherin-like transmembrane glycoproteins that are major constituents of the desmosome.

Junctional adhesion molecule A	F11R	[6]	Involved in epithelial tight junction formation.
Purinergic receptor P2X 6	P2RX6	[14]	Associated with VE-cadherin at adherens junctions of human umbilical vein endothelial cells.
Tight-junction protein 1/ zonulin 1	TJP1	[14]	Acts as a tight junction adaptor protein that also regulates adherens junctions.
Vezatin/ adherens junctions transmembrane protein	VEZT	[14]	Transmembrane protein which has been localized to adherens junctions and shown to bind to myosin VIIA. The expression of VEZY is decreased in gastric cancer.

Table S2. Markers of M1 and M2 macrophages (for Fig. 5)

**Gene Name**	**Abbreviation**	**Ref.**	**M1/M2 specificity**
Scavenger receptor sys-rich type 1 protein M130	CD163	[29, 30]	M2
Macrosialin	CD68	[29, 30]	M1
T-lymphocyte activation antigen cd80	CD80	[29, 30]	M1
T-lymphocyte activation antigen cd86	CD86	[29, 30]	M1/ M2
MCH Class II Isotype	HLA-DMA	[29, 30]	M1/ M2
MCH Class II Isotype	HLA-DMB	[29, 30]	M1/ M2
MCH Class II Isotype	HLA-DOA	[29, 30]	M1/ M2
MCH Class II Isotype	HLA-DOB	[29, 30]	M1/ M2
MCH Class II Isotype	HLA-DPA1	[29, 30]	M1/ M2
MCH Class II Isotype	HLA-DPB1	[29, 30]	M1/ M2
MCH Class II Isotype	HLA-DQA1	[29, 30]	M1/ M2
MCH Class II Isotype	HLA-DQB1	[29, 30]	M1/ M2
MCH Class II Isotype	HLA-DRA	[29, 30]	M1/ M2
MCH Class II Isotype	HLA-DRB1	[29, 30]	M1/ M2
MCH Class II Isotype	HLA-DRB5	[29, 30]	M1/ M2
Interleukin-1 receptor type 1	IL1R1	[29, 30]	M1/ M2
Macrophage mannose receptor	MRC1	[29, 30]	M2
Macrophage scavenger receptor type 1	MSR1	[29, 30]	M2
Nitric oxide synthase (inducible)	NOS2	[29, 30]	M1
Suppressor of cytokine signaling 3	SOCS3	[29, 30]	M1
Toll-like receptor 2	TLR2	[29, 30]	M1
Toll-like receptor 4	TLR4	[29, 30]	M1

Table S3. Genes associated with apoptosis and proliferation (for Fig. 8).

Gene Name	Abrrev.	Function	Citation
Bcl2-associated agonist of cell death	BAD	Pro-apoptotic proteins via intrinsic apoptosis.	[31]
Caspase 9	CASP9	Activates caspase cascades and executes apoptosis. Promotes ABL1/c-Abl-dependent DNA damage apoptosis.	
TP53 binding protein 1	TP53BP1	Involved in repairing DNA double-stranded breaks.	[32]
Tyrosine-protein kinase ABL1	ABL1	Pro-apoptotic in presence of severe DNA damage by phosphorylating CASP9. Regulates actin remodeling.	[33]
Cyclin-dependent kinase 1	CDK1	Overexpression inhibits apoptosis and drives cell proliferation and promotes survival.	[34]
Proliferating cell nuclear antigen	PCNA	Processivity factor in DNA synthesis, overexpression increases cell proliferation. Reducing PCNA can induce apoptosis. Known to have divergent properties in response to p53 mutation.	[35, 36]

1. Lee, H.W.; Park, Y.M.; Lee, S.J.; Cho, H.J.; Kim, D.-H.; Lee, J.-I.; Kang, M.S.; Seol, J.H.; Shim, Y.M.; Nam, D.-H.; et al. Alpha-Smooth Muscle Actin (ACTA2) Is Required for Metastatic Potential of Human Lung Adenocarcinoma. *Clin. Cancer Res.* **2013**, *19*, 5879–5889, doi:10.1158/1078–0432.CCR-13–1181.
2. Ding, L.; Zhang, Z.; Shang, D.; Cheng, J.; Yuan, H.; Wu, Y. Song, X. α-Smooth muscle actin-positive myofibroblasts, in association with epithelial-mesenchymal transition and lymphogenesis, is a critical prognostic parameter in patients with oral tongue squamous cell carcinoma. *J. Oral. Pathol. Med.* **2014**, *43*, 335–343, doi:10.1111/jop.12143.
3. Sudo, H.; Tsuji, A.B.; Sugyo, A.; Abe, M.; Hino, O.; Saga, T. AHNAK is highly expressed and plays a key role in cell migration and invasion in mesothelioma. *Int. J. Oncol.* **2014**, *44*, 530–538, doi:10.3892/ijo.2013.2183.
4. Chang, H.; Liu, Y.; Xue, M.; Liu, H.; Du, S.; Zhang, L.; Wang, P. Synergistic action of master transcription factors controls epithelial-to-mesenchymal transition. *Nucleic Acids Res.* **2016**, *44*, 2514–2527, doi:10.1093/nar/gkw126.
5. Zhang, L.; Ye, Y.; Long, X.; Xiao, P.; Ren, X.; Yu, J. BMP signaling and its paradoxical effects in tumorigenesis and dissemination. *Oncotarget* **2016**, *7*, 78206–78218, doi:10.18632/oncotarget.12151.
6. Bateman, A.; Martin, M.J.; O'Donovan, C.; Magrane, M.; Alpi, E.; Antunes, R. UniProt: The universal protein knowledgebase. *Nucleic Acids Res.* **2017**, *45*, D158–D169, doi:10.1093/nar/gkw1099.
7. Lamouille, S.; Xu, J.; Derynck, R. Molecular mechanisms of epithelial-mesenchymal transition. *Nat Rev Mol Cell Biol.* **2014**; 15:178–96, doi:10.1038/nrm3758.
8. Thiery, J.P.; Acloque, H.; Huang, R.Y.J.J.; Nieto, M.A. Epithelial-Mesenchymal Transitions in Development and Disease. *Cell*, **2009**, *139*, 871–90, doi:10.1016/j.cell.2009.11.007.
9. Parsana, P.; Amend, S.R.; Hernandez, J.; Pienta, K.J.; Battle, A. Identifying global expression patterns and key regulators in epithelial to mesenchymal transition through multi-study integration. *BMC Cancer* **2017**, *17*, 447, doi:10.1186/s12885–017–3413–3.
10. Praveen Kumar, V.R.; Sehgal, P.; Thota, B.; Patil, S.; Santosh, V.; Kondaiah, P. Insulin like growth factor binding protein 4 promotes GBM progression and regulates key factors involved in EMT and invasion. *J. Neurooncol.* **2014**, *116*, 455–464, doi:10.1007/s11060–013–1324-y.
11. Argraves, W.S.; Pytela, R.; Suzuki, S.; Millán, J.L.; Pierschbacher, M.D.; Ruoslahti, E. cDNA sequences from the α

subunit of the fibronectin receptor predict a transmembrane domain and a short cytoplasmic peptide. *J. Biol. Chem.* **1986**, *261*, 12922–12924.

12. Dravis, C.; Spike, B.T.; Harrell, J.C.; Johns, C.; Trejo, C.L.; Southard-Smith, E.M.; Perou, C.M.;Wahl, G.M. Sox10 Regulates Stem/Progenitor and Mesenchymal Cell States in Mammary Epithelial Cells. *Cell Rep.* **2015**, *12*, 2035–2048, doi:10.1016/j.celrep.2015.08.040.
13. Lili, L.N.; Matyunina, L.V.; Walker, L.D.; Wells, S.L.; Benigno, B.B.; McDonald, J.F. Molecular profiling supports the role of epithelial-to-mesenchymal transition (EMT) in ovarian cancer metastasis. *J. Ovarian Res.* **2013**, *6*, 49, doi:10.1186/1757–2215–6-49.
14. O'Leary, N.A.; Wright, M.W.; Brister, J.R.; Ciufo, S.; Haddad, D.; McVeigh, R.; Rajput, B.; Robbertse, B.; Smith-White, B.; Ako-Adjei, D.; et al. Reference sequence (RefSeq) database at NCBI: Current status, taxonomic expansion, and functional annotation. *Nucleic Acids Res.* **2016**, *44*, D733–D745, doi:10.1093/nar/gkv1189.
15. Xiang, J.; Hu, Q.; Qin, Y.; Ji, S.; Xu, W.; Liu, W.; Shi, S.; Liang, C.; Liu, J.; Meng, Q.; et al. TCF7L2 positively regulates aerobic glycolysis via the EGLN2/HIF-1α axis and indicates prognosis in pancreatic cancer. *Cell Death Dis.* **2018**, *9*, 321, doi:10.1038/s41419–018–0367–6.
16. D'Angelo, R.C.; Liu, X.-W.; Najy, A.J.; Jung, Y.S.; Won, J.; Chai, K.X.; Fridman, R.; Choi Kim, H. TIMP-1 via TWIST1 Induces EMT Phenotypes in Human Breast Epithelial Cells. *Mol. Cancer Res.* **2014**, *12*, 1324–1333, doi:10.1158/1541–7786.MCR-14–0105.
17. Tadokoro, A.; Kanaji, N.; Liu, D.; Yokomise, H.; Haba, R.; Ishii, T.; Takaji, T.; Watanabe, N.; Kita, N.; Kadowaki, N.; et al. Vimentin Regulates Invasiveness and Is a Poor Prognostic Marker in Non-small Cell Lung Cancer. *Anticancer Res.* **2016**, *36*, 1545–1551.
18. Mendez, M.G.; Kojima, S.-I.; Goldman, R.D. Vimentin induces changes in cell shape, motility, and adhesion during the epithelial to mesenchymal transition. *FASEB J.* **2010**, *24*, 1838–1851, doi:10.1096/fj.09–151639.
19. Reya, T.; Clevers, H. Wnt signalling in stem cells and cancer. *Nature* **2005**, *434*, 843–850, doi.org/10.1038/nature03319.
20. Shen, X.; Kramer, R.H. Adhesion-Mediated Squamous Cell Carcinoma Survival through Ligand-Independent Activation of Epidermal Growth Factor Receptor. *Am. J. Pathol.* **2004**, *165*, 1315–1329, doi:10.1016/S0002–9440(10)63390–1.
21. Yang, J.; Hou, Y.; Zhou, M.; Wen, S.; Zhou; J.; Xu, L.; Tang, X.; Du, Y.-E.; Hu, P.; Liu, M. Twist induces epithelial-mesenchymal transition and cell motility in breast cancer via ITGB1-FAK/ILK signaling axis and its associated downstream network. *Int. J. Biochem. Cell Biol.* **2016**, *71*, 62–71, doi:10.1016/j.biocel.2015.12.004.
22. Lin, J.; Myers, A.L.; Wang, Z.; Nancarrow, D.J.; Ferrer-Torres, D.; Handlogten, A.; Leverenz, K.; Bao, J.; Thomas, D.G.; Wang, T.D.; et al. Osteopontin (OPN/SPP1) isoforms collectively enhance tumor cell invasion and dissemination in esophageal adenocarcinoma. *Oncotarget* **2015**, *6*, 22239–22257, doi:10.18632/oncotarget.4161.
23. Guo, X.; Xu, Y.; Zhao, Z. In-depth genomic data analyses revealed complex transcriptional and epigenetic dysregulations of BRAF V600E in melanoma. *Mol. Cancer* **2015**, *14*, 1–9, doi:10.1186/s12943–015–0328-y.
24. Chowdhury, R.; Webber, J.P.; Gurney, M.; Mason, M.D.; Tabi, Z.; Clayton, A. Cancer exosomes trigger mesenchymal stem cell differentiation into pro-angiogenic and pro-invasive myofibroblasts. *Oncotarget* **2015**, *6*, 715–731, doi:10.18632/oncotarget.2711.
25. Núñez-Wehinger, S.; Ortiz, R.J.; Díaz, N.; Díaz, J.; Lobos-González, L.; Quest, A.F.G. Caveolin-1 in cell migration and metastasis. *Curr. Mol. Med.* **2014**, *14*, 255–274, doi:10.2174/1566524014666140128112827.
26. Yamasaki, T.; Seki, N.; Yoshino, H.; Itesako, T.; Hidaka, H.; Yamada, Y.; Tatarano, S.; Yonezawa, T.; Kinoshita, T.; Nakagawa, M.; Enokida, H. MicroRNA-218 Inhibits Cell Migration and Invasion in Renal Cell Carcinoma through Targeting Caveolin-2 Involved in Focal Adhesion Pathway. *J. Urol.* **2013**, *190*, 1059–1068, doi:10.1016/j.juro.2013.02.089.
27. Ohashi, S.; Natsuizaka, M.; Wong, G.S.; Michaylira, C.Z.; Grugan, K.D.; Stairs, D.B.; Kalabis, J.; Vega, M.E.; Kalman, R.A.; Nakagawa M; et al. Epidermal growth factor receptor and mutant p53 expand an esophageal cellular subpopulation capable of epithelial-to-mesenchymal transition through ZEB transcription factors. *Cancer Res.* **2010**, *70*, 4174–4184, doi:10.1158/0008–5472.CAN-09–4614.
28. Xu, J.; Lamouille, S.; Derynck, R. TGF-beta-induced epithelial to mesenchymal transition. *Cell Res.* **2009**, *19*, 156-72, doi:10.1038/cr.2009.5.
29. Rőszer, T.; Corporation, H.P. Understanding the Mysterious M2 Macrophage through Activation Markers and Effector Mechanisms. *Mediators Inflamm.* **2015**, *2015*, 816460, doi:10.1155/2015/816460.
30. Duluc, D.; Delneste, Y.; Tan, F.; Moles, M.; Grimaud, L.; Lenoir, J.; Preisser, L.; Anegon, I.; Catala, L.; Ifrah, N.; et al. Tumor-associated leukemia inhibitory factor and IL-6 skew monocyte differentiation into tumor-associated-

macrophage-like cells. *Blood* **2007**, *110*, 4319–4331, doi:10.1182/blood-2007–02–072587.
31. Kiraz, Y.; Adan, A.; Kartal Yandim, M.; Baran, Y. Major apoptotic mechanisms and genes involved in apoptosis. *Tumor Biol.* **2016**, *37*, 8471–8486, doi:10.1007/s13277–016–5035–9.
32. Callen, E.; Di Virgilio, M.; Kruhlak, M.J.; Nieto-Soler, M.; Wong, N.; Chen, H.T.; Faryabi, R.B.; Polato, F.; Santos, M.; Stranes, L.M.; et al. 53BP1 mediates productive and mutagenic DNA repair through distinct phosphoprotein interactions. *Cell* **2013**, *153*, 1266–1280, doi:10.1016/j.cell.2013.05.023.
33. Wang, J.Y. Regulation of cell death by the Abl tyrosine kinase. *Oncogene* **2000**, *19*, 5643–5650, doi:10.1038/sj.onc.1203878.
34. Liu, P.; Kao; T.P.; Huang, H. CDK1 promotes cell proliferation and survival via phosphorylation and inhibition of FOXO1 transcription factor. *Oncogene* **2008**, *27*, 4733–4744, doi:10.1038/onc.2008.104.
35. Fukami-Kobayashi, J.; Mitsui, Y. Overexpression of proliferating cell nuclear antigen in mammalian cells negates growth arrest by serum starvation and cell contact. *Jpn. J. Cancer Res.* **1999**, *90*, 286–293, doi:10.1111/j.1349–7006.1999.tb00746.x.
36. Paunesku, T.; Mittal, S.; Protić, M.; Oryhon, J.; Korolev, S.V.; Joachimiak, A.; Woloschak, G.E. Proliferating cell nuclear antigen (PCNA): Ringmaster of the genome. *Internat. J. Radiat. Biol.* **2001**, *77*, 1007–1021, doi:10.1080/09553000110069335.
